# Five Degree Internal Conical Connection and Marginal Bone Stability around Subcrestal Implants: A Retrospective Analysis

**DOI:** 10.3390/ma13143123

**Published:** 2020-07-13

**Authors:** Diego Lops, Michele Stocchero, Jason Motta Jones, Alessandro Freni, Antonino Palazzolo, Eugenio Romeo

**Affiliations:** 1 Department of Prosthodontics, Dental Clinic, School of Dentistry, University of Milan, 20142 Milan, Italy; antonino.palazzolo@unimi.it (A.P.); eugenio.romeo@unimi.it (E.R.); 2 Department of Neurosciences, University of Padova, 35128 Padua, Italy; stocchero.michele@gmail.com; 3 Department of Oral Surgery, Dental Clinic, Humanitas University, 20089 Milan, Italy; motta@studiomottarossi.it; 4 Private Practice, 22030 Orsenigo, Italy; info@freniorsenigo.it

**Keywords:** dental implant, marginal bone loss, retrospective studies, subcrestal implant placement

## Abstract

Background: There is limited information on the effect of the connection between subcrestally placed implants and abutments on marginal bone levels. The aim of the present retrospective study was to evaluate marginal bone levels after definitive prosthesis delivery around implants with an internal 5° conical connection placed in a subcrestal position. Materials and methods: Patients treated with fixed prostheses supported by implants placed at a subcrestal level between 2012 and 2018 were recalled for a follow-up examination. All implants had 5° internal conical connection with platform switching. Radiographic marginal bone level (MBL) was measured. MBL change between prosthetic delivery (t_0_) and follow-up examination (t_1_) was calculated. A multiple regression model was performed to identify the most significant predictors on MBL change. Results: Ninety-three patients and 410 implants, with a mean follow-up of 2.72 ± 1.31 years, were examined. Mean MBL was −1.09 ± 0.65 mm and −1.00 ± 0.37 mm at t_0_ and t_1_, respectively, with a mean bone remodeling of 0.09 ± 0.68 mm. An implant’s vertical position in relation to the bone crest, the year of follow up and the presence of type-2 controlled diabetes were demonstrated to be influencing factors for MBL modifications. Conclusions: Subcrestally placed implants with platform switching and internal conical connection maintained stable bone levels over a mean follow-up of more than 2 years. How a tight internal conical connection between abutment and implant may contribute to this clinical evidence should be more deeply investigated. MBL variations seem to be mostly influenced by an implant’s vertical position and presence of type-2 controlled diabetes.

## 1. Introduction

The use of dental implants has become a common option for the fixed rehabilitation of single, multiple, and complete edentulous areas [1,2]. Long-term stability of marginal bone level (MBL) around implants is one of the most commonly used criteria for defining implant success [3]. According to previous reports on two-piece implants, marginal bone resorption mostly occurs within the first year of implant functioning [4,5]. After this initial crestal alteration, an implant typically develops a steady state situation with no relevant further bone loss in the following years [6]. Nevertheless, for an optimal result, preserving the initially-achieved marginal bone levels to be as coronal as possible has been advocated for [7].

From the treatment protocol proposed by Brånemark, several modifications have been introduced over the past decades [8,9]. Different implant macro and micro designs, surgical procedures, and prosthetic protocols have been developed in order to improve implant treatments’ functional and aesthetic outcomes [10,11]. More specifically, implant-abutment interface (IAI) design and location have been deeply examined to minimize early marginal bone resorption [12,13,14]. It was observed that internal tapered IAI may provide better results in terms of stability, seal performance, and better load distribution compared to butt-joint interfaces [15,16,17]. Another strategy was to generate a horizontal discrepancy in diameter between the implant and the abutment, namely platform-switching [18]. Such an approach may contribute to preserving crestal bone levels [13].

Conical connections have proven to be superior to butt-joint interfaces at achieving a tight seal and reducing the microgap at the IAI, and have demonstrated improvements in crestal bone maintenance [19]. Nevertheless, most of clinical data on this type of connection are in reference to bone level implants [19]. A further factor that may influence the stability of peri-implant tissues is the IAI vertical position according to the bone ridge [20]. The surgical placement of the implant below the level of the crestal bone has been proposed to increase the esthetic emergence profile and avoid the implant threads’ exposure after the initial bone remodeling [21,22,23]. Peri-implant tissue alterations around implants placed subcrestally after 12 and 18 months were evaluated by some recent clinical trials [24,25]. No influence of implant vertical positioning was found. However, data on marginal bone maintenance for implants placed in a subcrustal position is actually limited, and the effect of the type of conical seal between abutment and fixture is still controversial.

Therefore, the aim of the present retrospective study was to assess marginal bone levels after definitive prosthesis delivery around implants with an internal 5° conical connection placed in a subcrestal position.

## 2. Materials and Methods

### 2.1. Patient Selection

The protocol of the present human study was approved by the University of Milan Ethical Committee (Prot. No. EC 02.04.20 REF 28/20) and was in accordance with the Helsinki Declaration guidelines, as revised in Fortaleza (2013).

**Figure 1:**
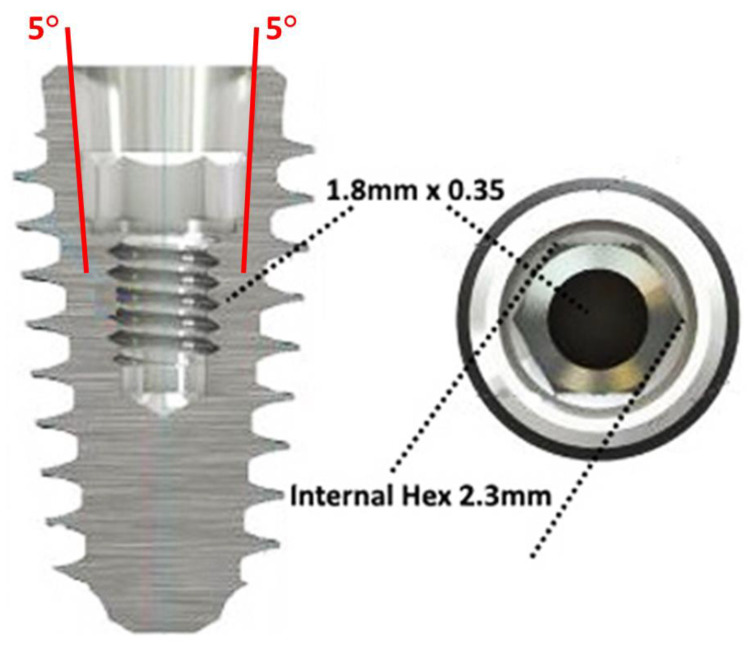
Schematic view of the implant system adopted in this study. Details of the 5 degree internal tapered IAI (implant abutment interface) are shown: the internal hexagon index was 2.3 mm wide, while the internal screw canal measured 1.8 × 3.5 mm.

All patients consecutively referred to a single dental clinic (Department of Prosthodontics, University of Milan, Milan, Italy) and treated with implant-supported fixed prostheses between January 2012 and December 2018 were screened for inclusion in this retrospective study. To be included in the study, all cases must have been treated with the same implant system provided with a 5 degree internal tapered IAI (Figure 1) and platform-switched abutments (Anyridge, MegaGen Implant Co., Gyeongbuk, South Korea) and placed at least 1 mm subcrestally.

Additional inclusion criteria were: absence of local inflammation; absence of oral mucosal disease; adequate oral hygiene; adequate bone volume at the implant site (enough for placement of implant at least 3.5 mm in diameter and 8 mm in length); and final rehabilitation with fixed implant-supported prosthesis.

Cases in which bone augmentation was necessary, or cases of immediate loading, were excluded. Furthermore, cases with no complete radiographical documentation from the implant placement to the follow-up control visit were excluded. 

Additional exclusion criteria were: patients with systemic diseases (such as heart, coagulation, and leukocyte diseases or metabolic disorders); history of radiation therapy in the head and neck region; current treatment with steroids; neurological or psychiatric handicap that could interfere with good oral hygiene; immuno-compromised status, including infection with human immunodeficiency virus; severe clenching or bruxism; smoking habit (more than 15 cigarettes per day); drug or alcohol abuse; and inadequate compliance.

All included patients gave their written consent after being informed in detail about the objectives of the study. Surgical and prosthetic procedures were performed by a single operator (AF), as described in the following paragraphs.

### 2.2. Surgical and Prosthetic Procedures

All implants were placed with a two-stage surgical protocol. Implants with internal conical connection were inserted by keeping the vertical position of the IAI 1–2 mm below the nominal crestal level, as recommended by the manufacturer. An intra-operative radiograph was taken to confirm the subcrestal position of the implant. An inter-implant distance of 3 mm at least, and/or an interproximal space from 1.5 to 3 mm between an implant and the adjacent tooth were observed [26,27,28,29,30]. Three months later, a surgical re-entry was performed and trans-mucosal healing abutments were connected to the implants.

Two weeks after surgical re-entry, an implant-level impression was taken for the fabrication of a one-piece ti-base screw-retained temporary prosthesis. Provisional restorations were inserted from one week to 6 weeks after implant level impression. After a further waiting period from 8 to 12 weeks for soft tissue conditioning, a definitive impression was taken.

Different types of fixed restoration were delivered, based on clinical indications: single crowns (SC), partial fixed dentures (FPD), full fixed dentures (FFD), or Toronto bridges (TO). For screw-retained prostheses, a torque of 25 Ncm was used to install the definitive restorations by means of a proper torque wrench. For cemented restorations, abutments were torqued down to 25 Ncm and restorations were luted with a temporary cement (Temp-Bond Clear, Kerr Corporation, OR, USA). At definitive prosthesis delivery (t_0_), an intraoral periapical radiograph was taken. 

### 2.3. Clinical and Radiographic Evaluations

All selected patients were recalled for a follow-up visit (t_1_) between September 2018 and February 2019. The following data were recorded: type of prosthesis, implant diameter and length, implant site, date of the prosthetic delivery, patient condition, including smoking habit (>10 cig/die), presence of type-2 diabetes, oral bisphosphonates drug intake. During the follow-up visit, an intraoral periapical radiograph and a clinical examination were performed. Furthermore, the peri-implant tissues and implant health status were evaluated following the guidelines on peri-implantitis and peri-implant mucositis diagnosis by the European Federation of Periodontology and the American Academy of Periodontology [31]. Peri-implant mucositis was defined if any bleeding and/or suppuration on gentle probing without bone loss. Besides, peri-implantitis was defined if any bleeding and/or suppuration on gentle probing, an increased probing depth and bone loss was detected.

All radiographs were taken with a standardized parallel technique with an X-ray apparatus supplied with a long cone and a Rinn Universal Collimator (Dentsply RINN, York, Pennsylvania) The following exposure parameters were used: 65-90 kV, 7.5-10 mA and 0.22-0,25 s. All radiographs were stored on a PC. Radiographic images were then analyzed with a software program (Image J, National Insitute of Health, Bethesda, Rockville, MA, USA). Before measurement, each radiograph was calibrated by using the implant diameter and length as reference measures to correct any distortion. 

**Figure 2:**
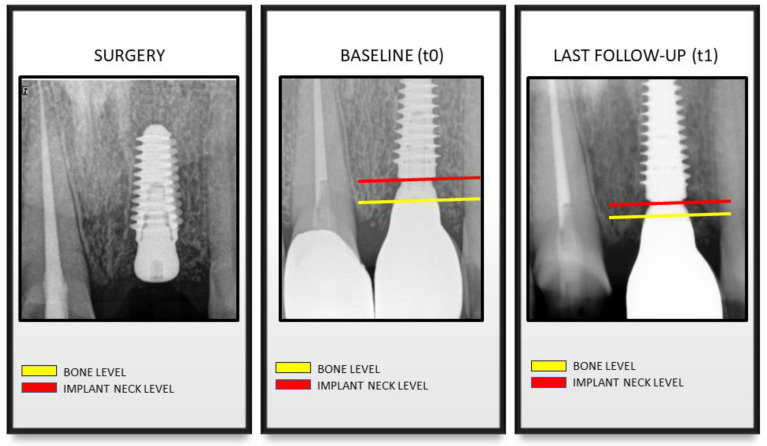
X-rays were taken at surgery, prosthetic delivery (baseline), and at the follow-up visit (t_1_). Measurements of marginal bone level (MBL) were taken for the mesial and the distal aspect of each implant.

Marginal Bone Level (MBL) was measured for t_0_ and t_1_ according to Linkevicius et al. as the following [32]: the distance between the implant neck (bevel) and the first bone-to-implant contact or the distance between the implant neck and the marginal bone in contact with the line drawn from the implant bevel following the long axis of the implant. Measurements were taken for the mesial and the distal aspect of each implant (Figure 2). An average value for each implant was then calculated. Measurements (in mm) were performed to the nearest 0.1 mm using a monitor with the screen resolution of 1920 x 1080 and a magnification (7x) of the images. A single operator performed all measurements (A.P.).

Measurements in which the implant neck was located below the bone level were classified as negative values. On the contrary, measurements in which implant neck was above the bone level were classified as positive values.

Based on the time elapsed between t_0_ and t_1_, cases were grouped by years of follow-up (2 years, 3 years, 4 years, 5 years, 6 years, 7 years and 9 years). 

### 2.4. Statistical Analysis 

Each implant was considered as the statistical unit. The following outcomes were considered: MBL, MBL change, implant survival, presence of mucositis, and presence of peri-implantitis. No significant differences were found by comparing the means of mesial and distal MBL at each follow-up with a *t*-test for paired data, hence the mean between mesial and distal MBL was used in the subsequent analysis. Descriptive statistics was performed by calculating mean and standard deviation for continuous variables and frequency distribution for categorical variables respectively. The normality of data distribution was checked. A univariate analysis on MBL change according to the effects of type of prosthesis, implant diameter, implant length, implant position, and jaw was performed using Kruskal-Wallis or Mann-Whitney U-tests. Moreover, the prosthesis type frequency distribution based on the follow-up was calculated. MBL change at patient level, using the patient as a unit was calculated as well. The influence of the patient’s clinical condition on MBL changes was verified using a Kruskal-Wallis test. Variables which showed a significant relationship with MBL change in a univariate analysis (MBL(t_0_) and pathology) were introduced into a step-wise multiple regression model, and the MBL change was used as the dependent variable. Major outliers were excluded, and the “pathology” outcome was transformed into “diabetes” (yes/no). A statistical software package (IBM SPSS Statistics for Mac, v. 22) was used for descriptive statistics and model processing. 

## 3. Results

### 3.1. Patient Features

Of all treated patients, 93 patients (44 males and 49 females) aged from 22 to 86 years (mean age 57.3 ± 34.4 years) were selected for the present study, according to the inclusion and exclusion criteria. 

A total of 410 implants were positioned and the average follow-up period was 2.72 years. Frequencies of implant diameter and length are reported in Table 1. Two-hundred thirty-one implants were inserted in the maxilla, while 179 in the mandible, respectively; fixture distribution in anterior or posterior area was reported in Table 2. The frequencies for each type of prosthesis are reported in Table 3.

### 3.2. Clinical and Radiographic Evaluations

Mean MBL was −1.09 ± 0.65 mm and −1.00 ± 0.37 mm at t_0_ and t_1_, respectively, with a mean marginal bone remodeling of 0.09 ± 0.68 mm (Table 4). MBL at t_1_ was significantly lower than MBL at t_0_ (*p* < 0.001, Wilcoxon-Mann. Whitney). There was a statistically significant difference on MBL change among the clinical conditions (*p* = 0.031, Kruskal-Wallis) (Table 5). Mean MBL change was greater in patients with diabetes (−0.42 ± 0.73 mm), however, 1 vs. 1 post-hoc tests did not reveal any difference between one condition and another. Mean values of MBL change at implants with 2 years of follow-up and implants with >2 years are showed in Table 6. Mean MBL change at implants with 2 years of follow-up was significantly lower than implants with >2 years (*p* < 0.001 Mann-Whitney U Test).

**Figure 3:**
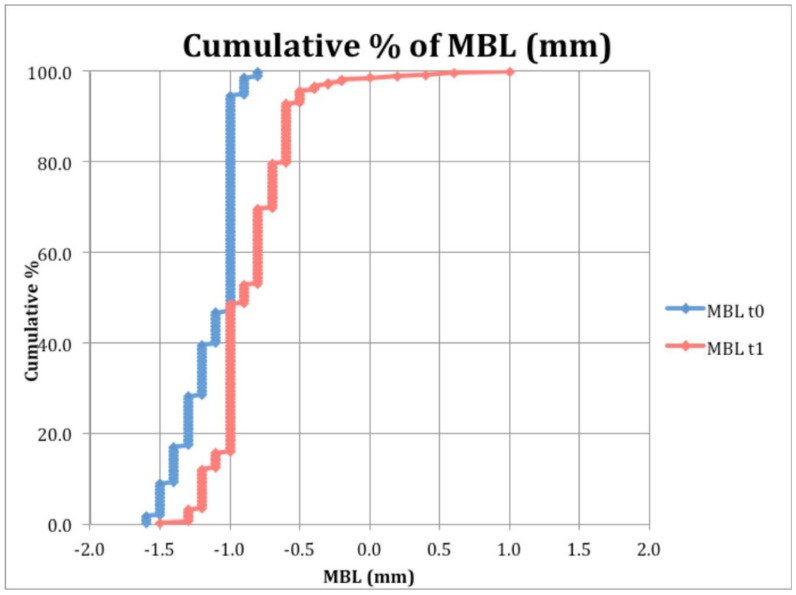
Cumulative percentage of MBL at baseline (t_0_) and after 2 years from loading.

Cumulative MBL at baseline (t_0_) and after 2 years from loading were calculated and are reported in Figure 3. 

Mean MBL change was also calculated for each patient based on clinical systemic conditions (Table 5, Table 6 and Table 7).

**Figure 4:**
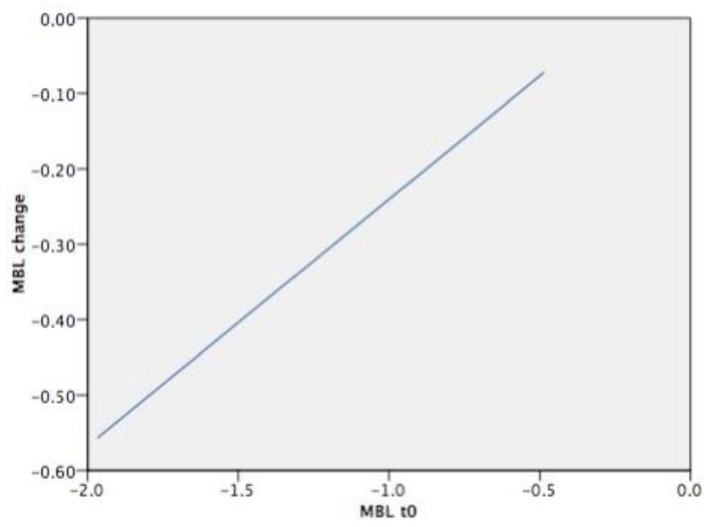
Estimated average MBL change according to MBL (t_0_). Results are in mm.

The multiple regression analysis revealed that the combination of time from prosthetic delivery (follow-up), the initial vertical position of the implant (MBL(t_0_)), and the presence of type-2 diabetes (Diabetes) may provide the highest predictability of MBL change (adjusted R2: 0.213). Follow-up resulted to be the most significant predictor for MBL change followed by the MBL(t_0_) and diabetes (Table 7). Estimated average values of MBL change according to the initial vertical position of the implant is shown in Figure 4, and according to the other predictors in Table 8.

## 4. Discussion

The aim of the present cohort study was to investigate the peri-implant marginal bone behavior around implants placed at subcrestal level in the medium to long-term period. Overall, the results showed that marginal bone alterations after definitive prosthesis delivery were modest during the follow-up period. On average, the implant neck was located 1.09 mm apical to the marginal bone level at time of definitive prosthesis delivery (t_0_). At follow-up examination, the average MBL was 1.00 mm apical to the marginal bone, and a clinically irrelevant bone loss of 0.09 mm was observed from t0 to t_1_. This finding is in accordance with previous studies showing that the major component of early marginal bone loss around both equicrestal and subcrestal implants occurs after implant uncovering and increases in the first months after functional loading before stabilizing [32,33,34]. In the present study, baseline radiography was performed at definitive prosthesis delivery (11–20 weeks after second stage surgery), when the greatest amount of bone remodeling due to the initial adaptation of the epithelial attachment and connective tissue compartment had already occurred. In fact, in a study on humans, Tomasi et al. retrieved biopsies around bone level implants and it was demonstrated that the thickness of the soft tissue barrier increased up to 3.6 mm until full tissue maturation, which occurred at 8 weeks from the abutment placement [35]. According to a recent meta-analysis, there are no relevant differences in bone loss between implants placed at subcrestal and equicrestal levels [36]. Likewise, the 1-year and 3-year outcomes of randomized clinical trials which compared implants placed 1.5 mm and 0.5 mm apical to the bone level, did not find any appreciable difference in marginal bone loss and clinical performance [25,37]. On the other hand, studies evaluating bone remodeling, rather than marginal bone loss, showed that implants placed subcrestally observed a greater extent of bone resorption during the first 12 month after prosthetic delivery [24,38]. Moreover, some clinical trials suggest that, in presence of thin supracrestal tissue, a subcrestal implant placement may reduce the probability for the implant to become exposed over time [39,40,41]. From the findings of the present study, it can be observed that at t_1_, the implant neck was located coronal to the marginal bone level only in very few cases. This indicates that the effect of the initial bone remodeling was limited. Such finding should be evaluated, since exposed treated surfaces may lead to plaque accumulation and inflammatory response, representing a potential factor for the onset of peri-implant inflammatory diseases [42]. Moreover, in the present report, a technical parameter, that is, the type of connection between the abutment and the fixture, was crucial for the achievement of clinical and radiographic outcomes during the entire follow-up period, as demonstrated in a recent meta-analysis concluding that crestal bone levels are better maintained in the short-medium term when implants with internal conical connections are used [43]. The multiple regression analysis performed in the present study aimed to identify the predictors for peri-implant bone loss. According to our findings, the most significant factors were implant vertical position and the presence of type-2 controlled diabetes. The MBL change was influenced by the initial vertical position of the implant at the time of prosthetic delivery. More in detail, the more apical the initial position of the implant, the greater the estimated bone loss value (Figure 2). This preliminary finding is of relevant clinical interest, since it would indicate which is the optimal position of the implant in relation to the marginal bone level. For implants placed 1 mm below the bone crest, no hard tissue remodeling was observed. For implants placed 2 or 3 mm below the bone level, a slight remodeling occurred, even though the implant neck was never exposed. It can be assumed that the conical connection as technical factor may help to achieve and maintain marginal bone level stability, but if the implant is placed too deeply in the bone, a bone remodeling will occur. This result is in agreement with a recent multicenter prospective clinical study showing a statistically significant positive correlation between the depth of implant insertion and early MBL [35]. Similarly, the present analysis suggested that the presence of type-2 controlled diabetes is also a predictor of bone loss. Such finding is in agreement with the current literature, since it has been established that implants in diabetic patients have a lower survival rate and a greater bone resorption than in non-diabetic patients [44,45]. However, the number of diabetic patients included in the present study (*n* = 4) was very low. The present results should be interpreted with caution, since a number of limitations are present. One limitation of this study was that the subgroups based on the follow-up were not equally distributed. The majority of included cases were analyzed after 2 years, and this might have influenced the overall results. Despite the promising outcomes on the peri-implant hard tissues’ stability, it can be concluded that more long-term data are required to confirm the clinical validity of implants placed at subcrestal level. Moreover, MBL was measured from the time of the definitive prosthetic delivery, while no information is available on the crestal alterations from the implant placement to the baseline. In this time frame, a number of different factors influence bone remodeling, including surgical trauma, biological width re-establishment, and number of abutment connection/disconnections. Another limitation is that the cases included in this analysis were restored with different kinds of prosthesis. Even though the influence of the type of rehabilitation on MBL change was not significant, a more comprehensive investigation on the prosthetic components is required in future studies. Finally, since only a single type of implant was selected in the analysis, the findings of the present study have to be interpreted cautiously when comparing different implant types. In fact, clinical and histological behavior of peri-implant hard and soft tissues in different implant platform surface configurations were studied by Monteiro et. al. [46]. Because marginal bone loss was less pronounced in roughened designs when compared to machined designs, it will be interesting to follow in time both machined and roughened implants with a 5° internal conical connection so that the real efficacy of such kinds of connections could be better understood. The effectiveness of a subcrestal implant placement has to be verified in long-term prospective clinical studies.

## 5. Conclusions

Within the limits of the present retrospective investigation, in a pool of subcrestally-placed implants, with 5 degree internal conical connection and a platform-switched abutment, MBL was stable. How a tight internal conical connection between abutment and implant may contribute to this clinical evidence should be more deeply investigated, due the fact that all implants had an internal connection. MBL change seems to be mostly influenced by the initial vertical position of the implant and the presence of type-2 controlled diabetes.

## Figures and Tables

**Table 1:** Frequency of implant length and implant diameter.

		Diameter (mm)					Total
		3.5	4	4.5	5	6.5	
**Length (mm)**	7	2	2	7	8	11	30
	8.5	4	13	1	2	12	32
	10	10	45	39	11	1	106
	11.5	11	8	3	1	0	23
	13	34	83	69	4	0	190
	15	22	3	4	0	0	29
**Total**		83	154	123	26	24	410

**Table 2:** Frequency of implant distribution by jaw and site of placement.

	Anterior	Posterior	Total
Maxilla	75	156	231
Mandible	45	134	179
Total	120	290	410

**Table 3:** Frequency of prosthesis type and respective follow-up time. SC: Single Crown; FPD: Fixed partial denture; FFD: Fixed full denture; TO: Toronto bridge.

Type of Prosthesis		Follow-Up							Total
		2 Years	3 Years	4 Years	5 Years	6 Years	7 Years	9 Years	
	SC	24	5	4	1	1	0	0	35
	FPD	55	11	14	11	1	3	1	96
	FFD	10	0	2	0	0	0	0	12
	TO	17	2	0	1	0	1	0	21
Total		106	18	20	13	2	4	1	164

**Table 4:** Mean marginal bone level (MBL) (mm) at implant insertion (t_0_) and at last follow-up visit (t_1_) and mean MBL change (mm) divided by time of follow-up. sd: Standard Deviation.

Follow-up	Frequency	MBL t_0_		MBL t_1_		MBL Change	
		Mean	sd	Mean	sd	Mean	sd
2 years	280	−1.09	0.37	−0.85	0.29	−0.24	0.41
3 years	38	−1.08	0.66	−1.02	0.39	−0.06	0.58
4 years	50	−0.77	1.32	−1.36	0.19	0.59	1.28
5 years	25	−1.62	0.20	−1.48	0.18	−0.14	0.29
6 years	3	−1.47	0.29	−1.30	0.17	−0.17	0.12
7 years	12	−1.12	1.31	−1.61	0.17	0.49	1.23
9 years	2	−2.10	0.14	−1.95	0.07	−0.15	0.21
Total	410	−1.09	0.65	−1.00	0.37	−0.09	0.68

**Table 5:** Mean MBL change (mm) calculated for each patient based on clinical systemic conditions. “No” stands for healthy patients with no clinical condition. sd: Standard Deviation.

		Clinical Conditions				
		No (*n* = 72)	Smoke (*n* = 15)	Diabetes (*n* = 4)	Bisphosphonates (*n* = 2)	*p* Value
MBL change	Mean	−0.13	−0.14	−0.42	1.30	0.031
	sd	0.49	0.14	0.25	1.98	

**Table 6:** MBL change 2 years vs. more than 2 years. sd: Standard Deviation.

	Vs. Followup	*n*	Mean	sd	*p* Value
MBL change	2 years	280	−0.24	0.41	*p* < 0.001
	>2years	130	0.22	0.99	

**Table 7:** Multivariate linear regression analysis with MBL change as the dependent variable (surgery to 5 years) and time from prosthetic delivery (follow-up), the initial vertical position of the implant (MBL(t_0_)), and the presence of type-2 diabetes (diabetes) as predictors.

Predictors	Unstandardized Coefficients (B)	Standard Error	t-Statistics	*p*-Value
Constant	0.062	0.052	1.195	0.233
Follow-up	0.086	0.011	8.165	<0.001
MBL(t_0_)	0.346	0.049	7.126	<0.001
Diabetes	−0.292	0.048	−6.033	<0.001

**Table 8:** Estimated MBL change (mm) according to years of follow-up and presence of diabetes.

	Predictor	MBL Change
Follow-up	2 years, 3 years	−0.43
	4 years, 5 years, 6 years, 7 years, 9 years	−0.21
Presence of diabetes	yes	−0.46
	no	−0.18
